# Curvature Fluctuations of Asymmetric Photopolymerized Networks: Impact of Solvent-Mediated Interfacial Exchanges

**DOI:** 10.1021/acsapm.6c00944

**Published:** 2026-07-10

**Authors:** Muhammad Ghifari Ridwan, Ghassan Sadaka, Mihai-Andru Angheliu, João T. Cabral

**Affiliations:** Department of Chemical Engineering, 4615 Imperial College London, London SW7 2AZ, United Kingdom

**Keywords:** frontal photopolymerization, asymmetric network, diffusion, spontaneous curvature, actuation

## Abstract

Spontaneous curvature fluctuations develop in asymmetric polymer networks fabricated by frontal photopolymerization (FPP) following “development”, the immersion into a selective solvent. We investigate the impact of the choice of solvent on the spatiotemporal response of the gradient networks, employing a model system of UV-cross-linked poly­(ethylene glycol) diacrylate. Experimentally, we find that FPP networks can remain planar or exhibit one or two curvature changes over time depending on the solvent. We quantify the solvent and monomer mass exchanges during development and the overall mass uptake or loss and find a correlation between solvent polarity and the propensity for curvature fluctuations. A simple FPP model accounting for interfacial diffusive mass transfer and the transient formation of asymmetric surface shrinkage and/or swelling boundary layers is found to account for all experimental data. Based on our results, we demonstrate a simple concept for the autonomous propulsion of a submerged device that exploits these spontaneous fluctuations with an appropriate solvent medium.

## Graphical abstract

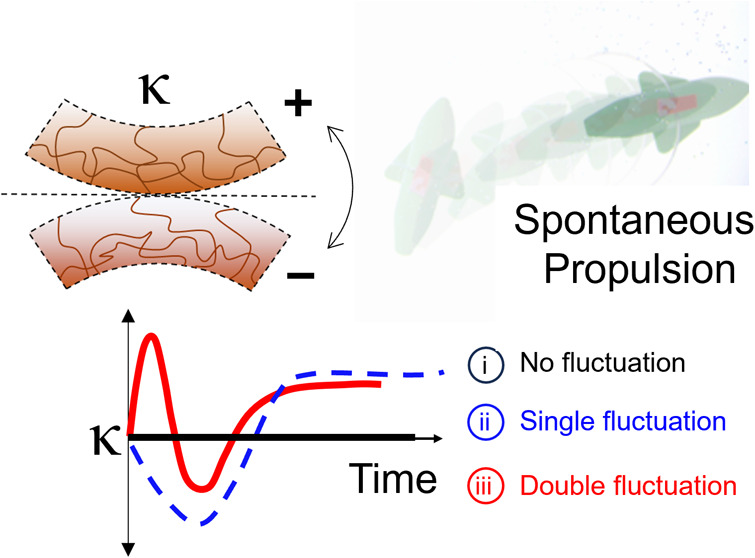

## Introduction

Tuning solvent–polymer interactions provides a powerful means to control stimuli-responsive behavior in functional polymer networks.^1–4^ Small differences in solvation and diffusivity can induce selective swelling, plasticization, or shrinkage, thereby modulating chain mobility and local mechanical properties.^5^ In 4D-printed architectures, these interactions are intentionally encoded through spatial variations in cross-linked density, polymer composition, or porosity, to enable predictable, time-dependent shape transformations under solvent exposure.^6–12^ Environmental actuation, employing solvents in the liquid or gas phase, can offer advantages over thermal or photochemical triggers, in terms of energy requirement, tunability, and compatibility with chemically diverse environments. By leveraging solvent gradients or uptake pathways, polymer networks can be engineered to exhibit complex spatial shape changes and adaptive responses.^13–15^

Such solvent-responsive behaviors have been integrated into soft robotic platforms that require compliant, environmentally adaptive, and autonomous actuation mechanisms.^9,16,17^ Hydrogels, elastomers, and shape-memory polymers engineered for selective solvent interactions can undergo reversible processes such as bending, contraction, or volumetric expansion, enabling complex motions with minimal mechanical hardware.^18,19^ Moreover, these same solvent–polymer principles can be harnessed to create embedded sensing elements that transduce chemical exposure into measurable electrical, optical, or mechanical signals.^14,20^ For example, variations in swelling strain, dielectric properties, or fluorescence intensity can serve as environmental sensors of solvent type, concentration, or diffusion rate.^14^ When combined with 4D-printed architectures, such integrated actuators and sensors allow soft robots to not only move in response to solvent cues but also monitor and interpret their chemical environment. This convergence of solvent-responsive materials, programmable manufacturing, and embedded sensing provides a powerful pathway toward multifunctional soft robotic systems capable of autonomous operation in complex or chemically dynamic settings.

Frontal photopolymerization (FPP) innately produces structures with tunable mechanical responses through the encoding of prescribed cross-linking gradients^21,22^ and definite spatiotemporal response during deswelling.^23,24^ This capability presents an opportunity to directly correlate molecular-scale network properties, such as monomer-to-polymer conversion and optical attenuation, with macroscopic performance metrics, such as modulus, strain, and spatiotemporal response.^23,25^ However, the interplay between the spatiotemporal evolution of asymmetric polymer networks and thermodynamic and kinetic properties of solvent environments and stimuli remains poorly understood.^23,24^ A rigorous mechanistic understanding of how this interplay influences the emergent mechanical behavior of gradient networks is essential for designing high-performance, adaptive materials.

Here, we present an experimental and theoretical investigation of asymmetric networks fabricated by FPP, examining the role of solvent immersion (intrinsic to the “development” of photopolymerized materials) in the evolution of pattern curvature. We seek to correlate solvent–polymer interactions and absorption capacity to curvature fluctuation. To interpret our data, we write a minimal model to describe the consequences of asymmetric network conversion, polymer absorption capacity, asymmetric solvent ingression and unreacted monomer extraction, and curvature fluctuations. Our experimental observations and coarse-grained model provide insights into the coupling between the network conversion profile, absorption capacity, diffusion, and stress distribution, establishing a framework to design and encode fluctuating curvature responses of asymmetric networks immersed in solvent media, paving the way for spontaneous actuation and propulsion.

## Experimental Section

### Chemicals

Photopolymerized networks were obtained from poly­(ethylene glycol) diacrylate (PEGDA, *M*_*n*_ = 575 g mol^–1^, Sigma-Aldrich, ref 437441) and the photoinitiator (PI) phenylbis­(2,4,6-trimethylbenzoyl)­phosphine oxide (Irgacure-819, BASF, ref 30128871). The photoinitiator-to-PEGDA mass ratio was fixed at 0.67%. Precursor formulations were prepared by mixing and stirring at 500 rpm for 1 h in a 30 mL glass vial wrapped in aluminum foil (to prevent inadvertent photopolymerization) and stored at 4 °C prior to use. Heptane, cyclohexane, toluene, isopropanol, ethanol (VWR Chemicals, 99.97%, ref 20821.321), acetone, ethylene glycol, methanol, *N*,*N*-dimethylformamide, water, and dimethyl sulfoxide were used as the developing solvent without further purification.

### Photopolymerization

The PEGDA/PI liquid mixture was placed between microscope glass slides (1 in ×3 in, Fisherbrand 1238–3118) and 1 mm-thick glass spacers at the edges. Photomasks of rectangles with prescribed dimensions were designed using AutoCAD 2024 and laser-printed on acetate films at 600 dpi. The masks were carefully placed onto the upper glass slide. UV irradiation was carried out using a collimated light source (Omnicure S1500) equipped with a 365 nm filter. The exposure irradiance was fixed within 2.0–2.5 mW cm^–2^ and measured before and after the experiment (VItec RS-365 digital radiometer, Spectroline) to calculate the exact UV exposure dose, accounting for the optical attenuation of the glass slides and photomask. An exposure dose of 18.9 mJ cm^–2^ was selected to fabricate PEGDA network films with a fixed *z*_f_ = 0.2 mm thickness, previously found to support curvature fluctuations.^12,26^ Following photopolymerization, PEGDA networks were removed from the glass substrate, pad-dried to remove monomer excess from the surfaces, and then immersed into various solvents for ‘development’. The film thickness was verified using a digital caliper (Mitutoyo, PK-0505CPX). Mass uptake measurements were carried out gravimetrically by sequentially immersing 7 mm × 2 mm × 0.2 mm beams into a given solvent and pad drying the solvent excess. Identical beams were mechanically clamped in the center and immersed within a solvent medium (∼75 mL) in a glass Petri dish (10 cm diameter) and imaged from below. Locally clamping at the beam center avoided pinning and depinning artifacts induced by surface contact that prevent accurate measurements of spontaneous curvature fluctuations. The beam response was imaged using a Basler acA640–750uc camera equipped with a 0.5–1× Edmund Optics lens (87,535), and data were analyzed with ImageJ.

### 3D-Printed Submersible

A submarine-shaped device was fabricated using a Bamboo Lab X1C 3D printer with poly­(lactic acid) (PLA) 1.75 mm filament. The geometry was designed by using SolidWorks 2023 and printed with a layer-by-layer thickness of 0.08 mm.

## Results and Discussion

### FPP Asymmetric Network Formation

Photopolymerization is a directional solidification process that converts a liquid monomer or prepolymer into a solid polymeric network. Under strong light attenuation and limited mass and thermal diffusion conditions, the network conversion profiles become progressively sharp, yielding planar traveling fronts that propagate along the direction of illumination, normal to the surface of the material (Figure 1a). This process is referred to as frontal photopolymerization, FPP, by analogy to autocatalytic frontal photopolymerization processes found in thermal or isothermal systems.^27^ The spatiotemporal evolution of FPP has been investigated experimentally and theoretically employing coarse-grained and explicit chemical models,^21,28–33^ examining the effect of optical attenuation, thermal and mass diffusion, reaction order, and precursor molecular mass. The directionality of the process encodes precise network conversion profiles, and associated physical, chemical, and mechanical properties, in the direction (*z*) normal to the surface of the patterned polymeric material, as illustrated in Figure 1a. Under photoinvariant conditions, or at low irradiation doses *d*, the network conversion profile ϕ­(*z*, *d*) of the traveling wave becomes effectively invariant

1 ϕ(z,d)=1−exp[−Kdexp(−μ̅z)]

where *K* is a conversion rate constant and μ̅ is the optical attenuation coefficient. In the unpolymerized state, ϕ≡0, becoming ϕ → 1 upon full polymerization. In this model framework, the position of the solid–liquid front *z*_f_, which defines the thickness of the solidified material, corresponds to the intersection of the ϕ­(*z*, *d*) profile with a solidification (or “percolation”) threshold ϕ_c_, yielding

2 $$z_{f}\left(d\right)=\frac{\ln\left[\frac{\mathrm{Kd}}{\ln\frac{1}{1-\phi_{c}}}\right]}{\mathrm{\mu ̅}}$$

**Figure 1:**
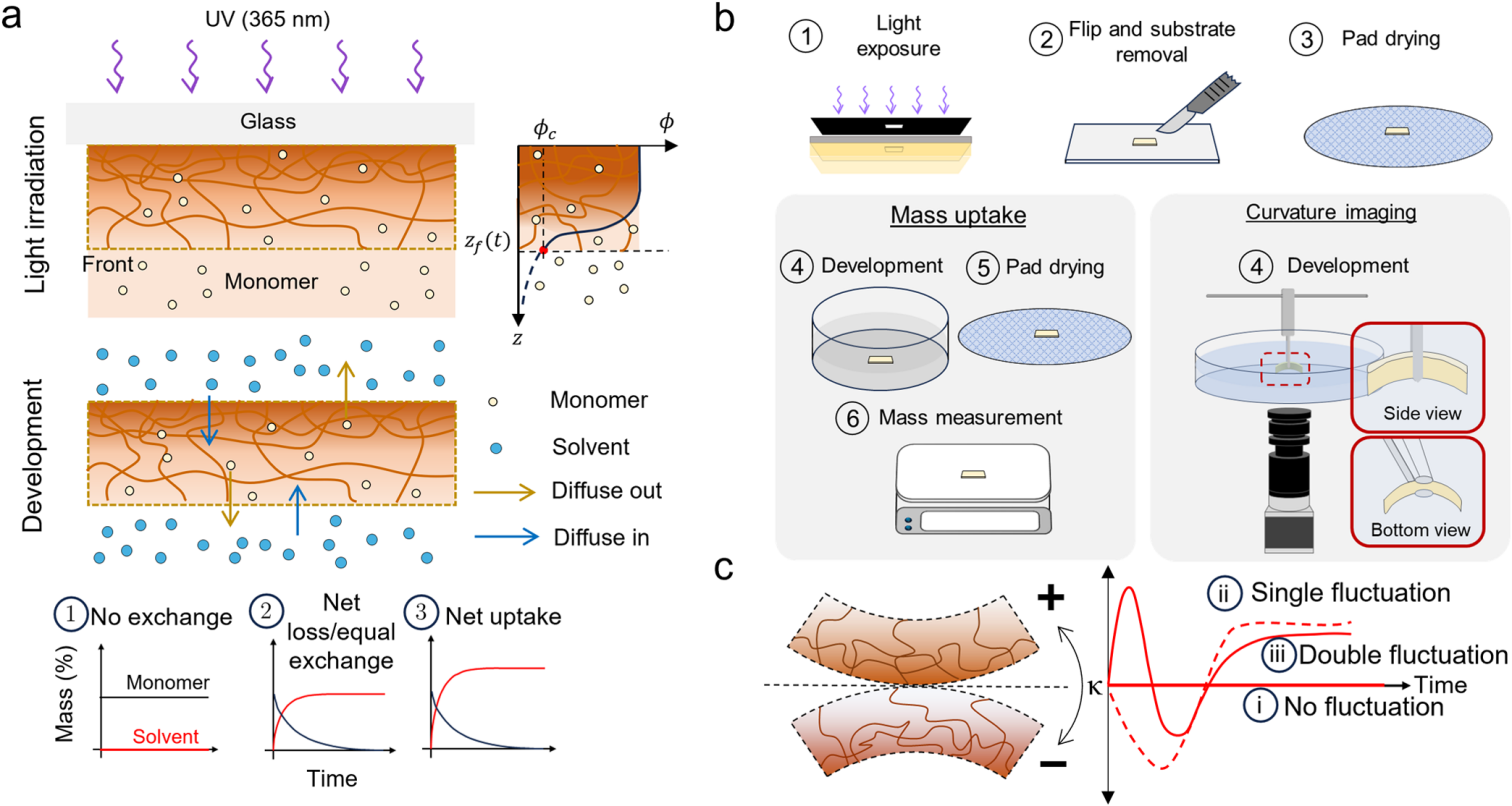
Frontal photopolymerization (FPP) of asymmetric PEGDA networks: interfacial exchanges during development and curvature fluctuations. (a) Fabrication of the asymmetric network via frontal photopolymerization, depicting the conversion profile ϕ­(*z*) and front position (*z*_f_), defining the patterned thickness at a given UV (365 nm) irradiation time (or dose, *d*). Development involves the interfacial exchange of the unreacted monomer and solvent, resulting in three possible scenarios: mass uptake, equal exchange, and net removal. (b) Schematic of FPP patterning (fabrication/light exposure, substrate removal, pad drying) and mass uptake experimental protocol or imaging of curvature evolution. (c) Schematic of asymmetric network curvature dynamics with three different outcomes: planar, single, and double fluctuations during solvent immersion (“development”).

In order to obtain a solid polymer material, the network is removed from the liquid medium and immersed or ‘developed’ into a selective solvent, to remove the excess monomer (and extract unreacted monomer or prepolymer). In our experiments, we employ poly­(ethylene glycol) diacrylate (PEGDA) as a model photopolymer system, as it is a widely used hydrophilic, biocompatible polymer with tunable mechanical properties and sorption capacity. Specifically, we select PEGDA with number-average mass *M_n_* = 575 g mol^–1^, parametrized by μ̅ = 3.4 mm^–1^ and *K* = 0.02 cm^2^ mJ^–1^ within the simplest “photoinvariant” FPP model.^26^ The profile asymmetry of PEGDA FPP networks was previously examined by FTIR spectroscopy by Vitale et al.^30^ and Zhao et al.^34^ using thicker polymer network sections. Since the focus of our paper is to examine and compare the role of the solvent media, we opt to fix the film thickness to *z*_f_ = 0.2 mm, obtained with an UV dose of *d* = 18.9 mJ cm^–2^ (corresponding to 10–15 s irradiance at 2.0–2.5 mW cm^–2^, as detailed in the Experimental Section). We select this thickness and conversion gradient as it was found to yield significant spatiotemporal response^12^ and repeatable fabrication. Evidently, both the film thickness *z*_f_ and conversion profile ϕ­(*z*) impact the magnitude and kinetics of curvature fluctuations and, for instance, sufficiently thick films can suppress fluctuation altogether;^26^ excessively thin films become difficult to manipulate, fragile, and tacky. In previous work, we have employed ethanol as the solvent “developer”.^12,26^ Here, we consider a range of polar and nonpolar candidate solvents, hypothesizing that solvent interfacial exchanges across otherwise identical asymmetric FPP networks can nontrivially impact the spatiotemporal response of the material curvature.

### Solvent–Polymer Polar Interaction Influences Absorption Capacity

We begin by examining the mass uptake of the PEGDA network with a series of representative solvents (Figures 1a and S1). We follow the experimental protocol depicted in Figure 1b: following light exposure, the gradient network is removed from the substrate, the excess prepolymer is pad-dried, and its mass was measured (comprising the polymer network and unreacted monomer); the network is then immersed for prescribed time intervals into the solvent, and the mass is measured as a function of immersion time. The mass changes for the unreacted monomer and solvent are then computed, as detailed in SI Section 1, considering repeatability and experimental uncertainty from separate experiments. In short, the total mass of the network + monomer + solvent is measured directly using a scale. At sufficiently long times, all monomer has exchanged with the solvent developer; drying of the solvent allows the exact mass of the polymer to be determined, and thus, the mass of the monomer + solvent at all times can be obtained. Given that the solvents are volatile, while the monomer is not (Figure S1), we measure the mass of the monomer (and thus that of the monomer too, by difference) as a function of immersion time, by evaporating the solvent at every time. Each data point in Figure 2a thus corresponds to an *individual* experiment (from photopolymerization to development at a fixed immersion time). Depending on the solvent, we visibly observe curvature fluctuations of the network beams, as illustrated in Figure 1c.

**Figure 2:**
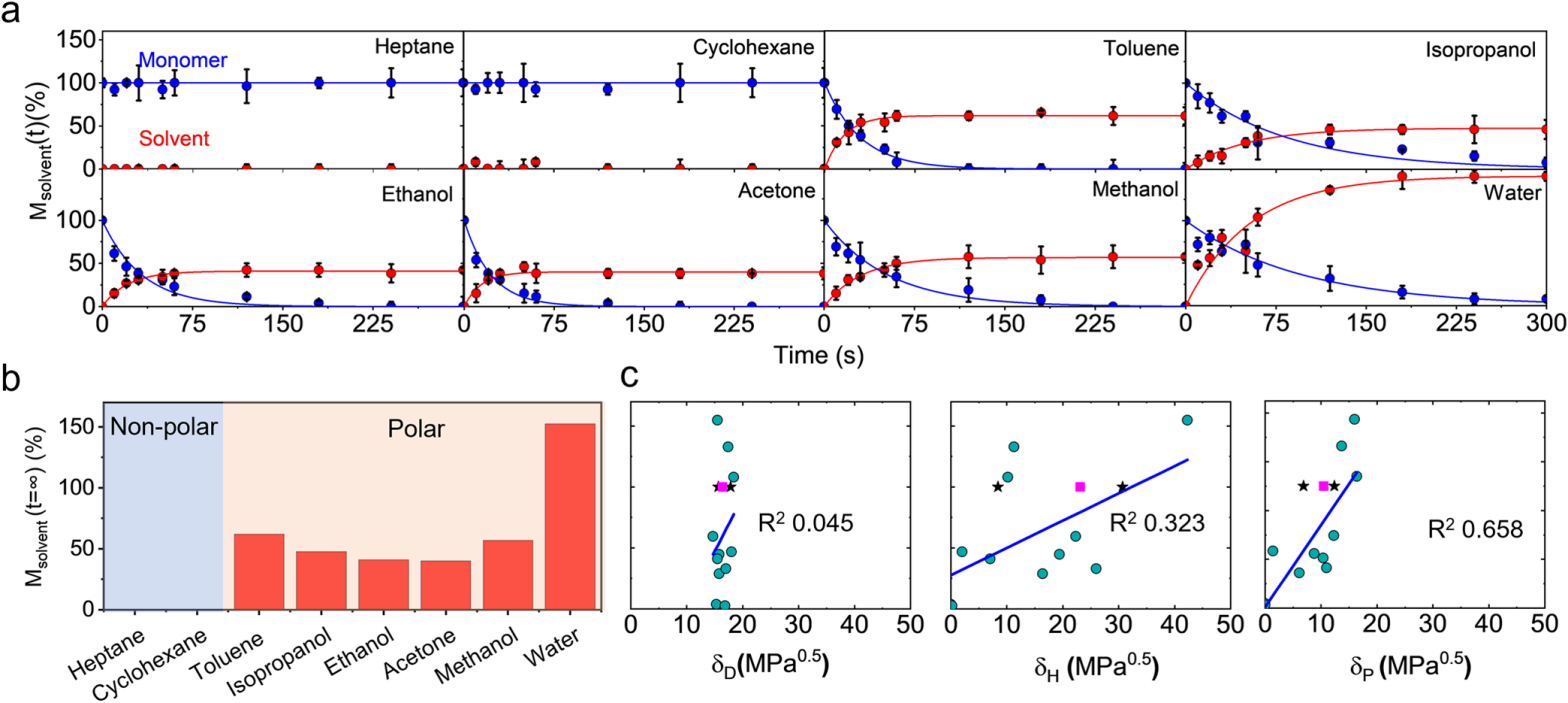
Mass uptake of the monomer and solvent in PEGDA asymmetric networks. (a) Measured mass uptake for selected solvents and the monomer in PEGDA asymmetric networks (*z*_f_ = 0.2 mm). Error bars were obtained from at least three repeated measurements. (b) Asymptotic solvent uptake (immersion *t* ≈ ∞) obtained experimentally. (c) Correlation between equilibrium solvent uptake (*t* ≈ ∞) with dispersive, hydrogen bond, and polar Hansen solubility parameters. Green circles indicate the solvents investigated, and the pink squares correspond to PEGDA, and black stars correspond to the center of two Hansen spheres for ethylene glycol and acrylate moieties (detailed in SI section 2). The blue solid line is a linear fit and corresponding *R* ^2^, suggesting a reasonable correlation with solvent polarity.

Figure 2a shows the mass change of the monomer and solvent in an asymmetric PEGDA network of thickness *z*_f_ = 0.2 mm. When the polymer is exposed to heptane and cyclohexane, there are effectively no mass changes in the monomer and solvent within the network. Immersion in toluene, isopropanol, ethanol, acetone, and methanol results in a decrease in monomer content (i.e., extraction of the unreacted monomer); the solvent content increases, and the overall net mass change is negative. Immersion in water results in strong solvent uptake as well as unreacted monomer extraction, yielding a positive net mass change.

While the sorption capacity of the network certainly depends on the photoconversion profile (ϕ­(*z*)) and precursor mass,^12,26^ this is fixed for this series of experiments, in order to isolate the role of the solvent “developer”. Expectedly, our experiments show marginal mass uptake by nonpolar solvents and considerable uptake of polar solvents, as shown in Figure 2b. We plot the equilibrium mass uptake *M*_total_ (*t* → ∞) of the cross-linked PEGDA as a function of the individual Hansen solubility parameters of the solvents, namely, dispersive δ_D_, polar δ_P_, and hydrogen bond δ_H_.^35^ For reference, an estimate of the parameters for PEGDA and ethylene glycol and acrylate moieties is also included (as detailed in SI Section 2). The highest correlation among all interaction parameters is found for the polar contribution δ_P_, albeit with a modest *R* ^2^ = 0.658 (Figure 2c).

### Emergence of Curvature (κ) Fluctuations of the Asymmetric Polymer Network in Solvent Immersion

Following photopolymerization, the polymer network is removed from the glass substrate and spontaneous curvature emerges. The curvature generally develops toward the less cross-linked network, i.e., toward decreasing ϕ­(*z*), which we define as negative curvature. The formation of spontaneous curvature of the asymmetric network is attributed to the differential shrinkage and mechanical heterogeneities of the polymer network.^23,34^

We then immerse the polymer network in various solvents mentioned above. In order to prevent pinning and depinning of the polymer network onto surfaces (e.g., of a container), we centrally clamp the polymer network beam to remain suspended during solvent immersion. Upon exposing the network with polar solvents (δ_P_ > 2 MPa^0.5^), we observe three distinct spatiotemporal responses of polymer networks (Figure 3). In the first instance (case ①), the curvature fluctuates by initially increasing in magnitude (dκ/d*t*_immersion_ > 0), reaching κ_max_, followed by a decrease in magnitude and change of sign (dκ/d*t*_immersion_ < 0), reaching κ_min_, and subsequently increasing (dκ/d*t*_immersion_ > 0) to reach κ_∞_ asymptotically. This behavior is observed in highly polar solvents, such as water and methanol. In water, κ_initial_ = 0.3 mm^–1^, followed by a sharp increase to κ_max_ = 1.1 mm^–1^, followed by a sharp decline to κ_min_ = 0.2 mm^–1^, and a gradual increase to κ_∞_ = 0.03 mm^–1^. For methanol, while it follows the same trend with water, the amplitude (*A* ≡ κ_max_ – κ_min_ in a fluctuating regime, detailed in SI Section 3) is lower, which is 0.5 mm^–1^. In case ②, the curvature immediately decrease in magnitude (dκ/d*t*_immersion_ < 0), reaching κ_min_, followed by an increase in magnitude and change of sign (dκ/d*t*_immersion_ > 0), reaching κ_∞_ asymptotically. This case can be represented by acetone, where κ_initial_ ≈ 0.3 mm^–1^ decreases to κ ≈ −0.25 mm^–1^ and increases again to κ_∞_ ≈ −0.1 mm^–1^. Isopropanol appears to exhibit a behavior of case ②, but we (reproducibly) find that κ_∞_ is positive and large, i.e., it curves pronouncedly toward the less cross-linked face. We hypothesize that such deviations can be linked to specific interactions with PEGDA via nonpolar and hydrogen bonding through its propane chain and alcohol functional groups, not captured by our coarse-grained model. In case ③, where we use a nonpolar solvent (δ_P_ < 2 MPa^0.5^), we observe monotonic curvature change upon immersion, such as heptane and cyclohexane. We observe a monotonic decrease of both κ, from κ_initial_ ≈ 0.1 mm^–1^ to reach κ_∞_ ≈ 0.25 mm^–1^.

**Figure 3:**
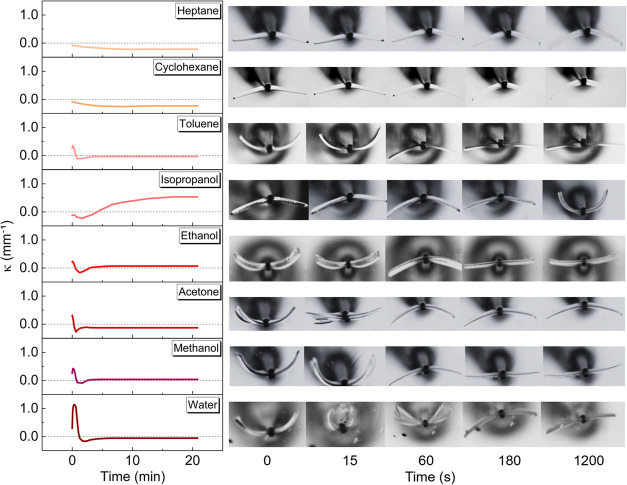
Spontaneous emergence of curvature of PEGDA asymmetric networks during immersion on different solvents. Evolution of beam curvature κ dynamic measurement results of asymmetric networks in different solvents along with the corresponding optical time-sequenced images of a beam (7 mm × 2 mm × *z*_f_ = 0.2 mm) held centrally by a mechanical clamp.

While we observe a clear correlation between solvent polarity and the amplitude of curvature fluctuations (SI Section 3), this should be interpreted as reflecting the broader consequences of the interplay between diffusion kinetics, swelling thermodynamics, and plasticization effects.^36–38^ Solvent uptake is governed by polymer–solvent compatibility,^38^ determined by the balance between mixing and elastic free energy contributions (see SI Section 2). In asymmetric networks, it is further modulated by the spatially varying cross-linking density. As a result, the local equilibrium swelling ratio varies across the network thickness, providing the structural basis for curvature generation.^37^ In parallel, the transient curvature response is controlled by the relative rates of solvent ingress and monomer egress, which in turn depends on molecular size (previously reported for this system^26^), intermolecular interactions, and local network structure, with more highly cross-linked regions restricting transport. As solvent sorption reduces the effective modulus, stress asymmetries are amplified during immersion. These coupled effects appear most pronounced for small, polar solvents that combine strong affinity with rapid diffusion, leading to pronounced and time-dependent curvature fluctuations. In contrast, nonpolar solvents exhibit negligible uptake, resulting in nearly invariant curvature, which is governed primarily by the initial conversion gradient. Based on this rationale, we developed a coarse-grained model for solvent actuation on asymmetric networks, described in the next section.

### Minimal Model for Solvent–Monomer Exchange in the Cross-Linked Asymmetric Network

To build a descriptive model to rationalize these curvature fluctuations under solvent immersion, we adapt a minimal simulation framework that accounts for a gradient cross-linking network, building upon Zhao et al.^23^ and our previous work.^12,26^ We extend the model by incorporating diffusion exchange phenomena between the solvent and monomer inside an asymmetric network. To capture the variability of absorption capacity of the polymer across different solvents, we carefully impose boundary conditions according to the mass uptake data for both monomer (*C*_m_(*z*, *t*_immersion_)) and solvent (*C*_s_(*z*, *t*_immersion_)) concentrations: (i) ∫_0_^*z*_f_^ *C*_m_(*z*, *t*_immersion_ = ∞)­ϕ^–1/3^d*z* = *M*_total,m_(*t*_immersion_ = ∞)/*A* and (ii) ∫_0_^*z*_f_^ *C*_s_(*z*, *t*_immersion_ = ∞)­ϕ^–1/3^d*z* = *M*_total,s_(*t*_immersion_ = ∞)/*A*, where *M*_total_, *A*, and *t*_immersion_ denote the total mass, surface area exposed to the solvent, and immersion time, respectively. The initial concentration profiles of the solvent and monomer, *C*_s_(*z*, *t* = 0) and *C*_m_(*z*, *t* = 0), respectively, are determined from experimental mass uptake data (*M*_total,s_(*t* = 0) and *M*_total,m_(*t* = 0); see Figure 2a). To establish the initial spatial distribution, we assume a power-law relation between the conversion ϕ­(*z*, *t*) and penetrant uptake by *M*(*t*_immersion_) = ∫0_*z*_f__ *C*_0_ϕ^–*n*^ from which the constant *C*_0_ is obtained. The initial concentration profile is therefore expressed as *C*_(*z*,0)_ = *C*_0_ϕ^–*n*^, where *n* is a fitting parameter representing the polymer absorption capacity (power-law exponent).

To couple the transport processes with the mechanical response of the network, we define a dimensionless swelling–shrinking parameter, the swell–shrink ratio, $\eta_{\mathrm{swell}-\mathrm{shrink}}\equiv \frac{C_{s}\left(z,t_{\mathrm{immersion}}\right)+C_{m}\left(z,t_{\mathrm{immersion}}\right)}{C_{m}\left(z,0\right)}$ . This ratio quantifies the relative change in total penetrant concentration with respect to the initial state. The network is considered to be shrinking when η_swell–shrink_ < 1 and swelling when η_swell–shrink_ > 1. An empirical constitutive relation linking Young’s modulus (*E*) and strain (ε) is introduced for the swelling and shrinking gradient network, expressed as functions of ϕ and η_swell–shrink_ (see SI Section S4). The mechanical evolution is evaluated by computing the time-dependent neutral axis of the beam

3 $$z_{N}\left(t_{\mathrm{immersion}}\right)=\frac{\int_{0}^{h}E\left(z,t_{\mathrm{immersion}}\right)\mathrm{zdz}}{\int_{0}^{h}E\left(z,t_{\mathrm{immersion}}\right)\mathrm{dz}}$$

and the corresponding curvature κ

4 $$\kappa \left(t_{\mathrm{immersion}}\right)=\frac{\int_{0}^{h}E\left(z,t_{\mathrm{immersion}}\right)\varepsilon \left(z-z_{N}\left(t_{\mathrm{immersion}}\right)\right)\mathrm{dz}}{\int_{0}^{h}E\left(t_{\mathrm{immersion}}\right){\left(z-z_{N}\left(t_{\mathrm{immersion}}\right)\right)}^{2}\mathrm{dz}}$$

In this framework, we rationalize the emergence of curvature fluctuations in terms of the variation of the position of the neutral axis and stress distribution over time, following asymmetric exchanges of the solvent and monomer at both top and bottom (high and low conversion, ϕ­(*z*)) interfaces. Further details of the minimal model, underlying assumptions, and limitations are provided in SI Section S4. We emphasize that our ‘minimal’ model is descriptive and interpretative in nature. In the SI, we also outline the requirements for a molecular-level framework that accounts for the explicit network thermodynamics, elasticity, and transport within a network of gradient mesh size.

### Asymmetric Diffusion Drives the Curvature Fluctuation

Conceptually, the mass transfer experienced by a polymer–monomer network immersed within a solvent could be classified according to ten possible scenarios, determined by the thermodynamics and kinetics of polymer–monomer–solvent ternary systems. These can be classified based on the diffusion rates of solvent ingress and monomer extraction into/from the polymer network, such that *D*_e,m_ > *D*_e,s_, *D*_e,m_ < *D*_e,s_, or *D*_e,m_ = *D*_e,s_, where *D*_e_ denotes an “effective” diffusion constant and subscripts m and s refer to the monomer and solvent, respectively, as well as based on the overall mass change of the polymer–monomer network upon solvent immersion: no exchange, net loss, equal exchange, or net gain. These various combinations define possible outcomes of the dynamic exchange between the monomer and solvent within a polymer network.

Based on our experimental observations, we consistently find the general trend *D*_e,m_ < *D*_e,s_, indicating that solvent diffusion is faster than “monomer” diffusion. This seems reasonable given the much larger mass of the PEGDA prepolymer (referred here as the effective “monomer” unit). Moreover, we do not observe cases of equal (and opposed) solvent and monomer that would result in a net zero mass change. We thus specify solvent network interactions into three distinct classes based on mass uptake behavior: ① no mass exchange (e.g., heptane), ② overall mass loss (e.g., ethanol), and ③ overall mass gain (e.g., water). The model parameters employed to simulate the curvature fluctuation of the asymmetric networks during solvent immersion are tabulated in SI Table S2. We first compute the overall mass uptake and η_shrink–swell_(*z*, *t*) distribution within the asymmetric network. For case ①, since there is no solvent ingression and no monomer extraction, the total mass does not increase (Figure 4a). Consequently, the distribution of solvent remains 0, and the monomer concentration profile remains unchanged from the initial conditions (SI Figure S4). This results in a constant η_shrink–swell_ throughout the time (Figure 4a). For case ②, the solvent ingression capacity is smaller than the monomer extraction capacity, resulting in overall shrinkage (Figure 4b). However, based on our mass update kinetic data, we note that the solvent ingression rate is faster than the monomer extraction rate. The solvent and monomer ingress and extraction across both interfaces result in the formation of “skin” layers that become solvent-rich, formed immediately after solvent immersion (*t* → 0^+^). As the process continues, the solvent ingress occurs deeper into the polymer bulk thickness, modeled assuming Fickian diffusion, reaching the solvent equilibrium, while the monomer is exchanged and drains out (SI Figure S5). Under the conditions investigated, the solvent ingression reaches the center of the film (*z*_f_ ≈ 0.1 mm) within 10 < *t* < 20 s. This is reflected on the overall shrinkage and results in a gradual decrease of η_shrink–swell_(<1) starting from the boundaries and slowly progresses into the polymer bulk (Figure 4b). By contrast, in case ③, the solvent sorption capacity and rate are both higher than the monomer extraction capacity and rate. This results in an overall mass uptake and swelling network (Figure 4c). As mentioned above, the solvent–monomer exchange at both interfaces results in the rapid formation of skin layers, and the solvent ingress reaches the network midpoint within *t* ≈ 5 s. Furthermore, the difference in solvent ingress and monomer extraction rate results in a transient out-of-equilibrium swollen state starting at *t* ≈ 50 s, as the mass uptake becomes greater than the equilibrium mass at long times. The processes continue and reach solvent equilibrium, while the monomer drains out. The overall swelling results in a gradual decrease of η_shrink–swell_ (>1) starting from the boundaries, slowly progressing into the polymer bulk (Figure 4c).

**Figure 4:**
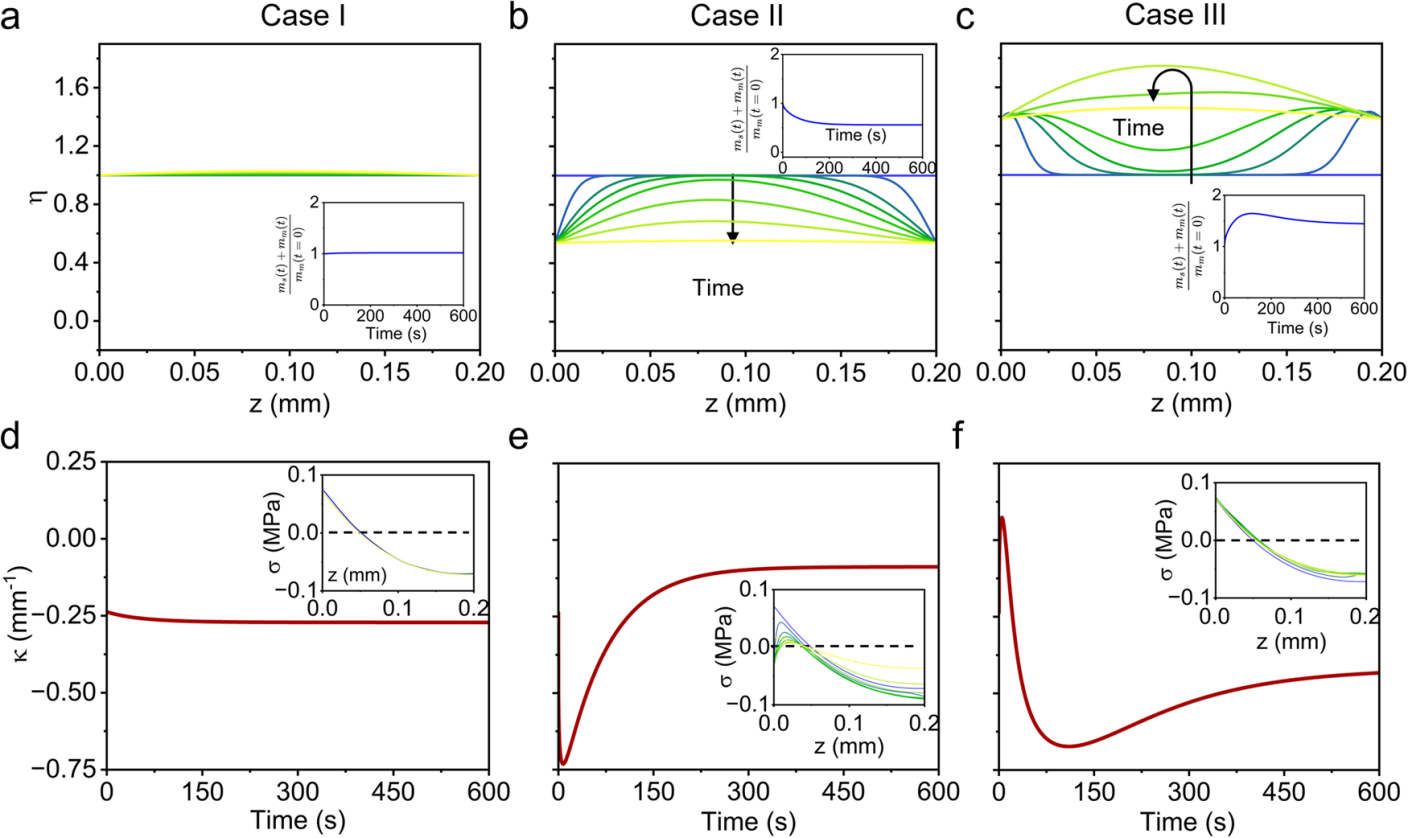
Computed spatiotemporal solvent–monomer distribution and mechanical properties across asymmetric networks. (a–c) Calculated η_swell–shrink_ ≡ (*C*_s_(*z*, *t*) + *C*_m_(*z*, *t*))/*C*_m_(*z*, 0), the ratio of evaporated solvent concentration to initial solvent concentration, across the polymer network (*z*_f_ = 0.2 mm) for three different representative solvents, heptane, ethanol, and water. Insets show the total mass change over time. (d–f) Simulated curvature dynamics for heptane, ethanol, and water, following the experimental observations shown in Figure 3. Insets show the computed stress distribution across the network thickness. The color gradient from blue to green to yellow indicates an increasing simulated time: 0, 1, 5, 10, 20, 50, 100, and 500 s.

We then simulate the implications of the asymmetry in network conversion and the rate and capacity of solvent ingression and monomer extraction to the mechanical response of the polymer network (Figure 4 and S5). In case ①, we expect the stress distribution to be generally governed by a tensile stress at *z* < *z*_N_ and a compressive stress at *z* > *z*_N_ (Figure 4d). The overall bending moment is thus negative, and hence, κ < 0 or close to zero. As there is no mass exchange, the stress distribution does not change over time, and hence, the κ profile remains invariant, i.e., there are no curvature fluctuations over time, as illustrated in Figure 4d. The encoded ϕ­(*z*) profile by the FPP process determines the (largely invariant) curvature. This behavior describes well solvents in case ①, such as heptane and cyclohexane.

When monomer and solvent exchanges take place, these are generally greater near the interface defined by the traveling front (fixed at *z*_f_ = 0.2 mm in both the experiment and simulation). The network conversion gradient breaks the symmetry of mass transfer at the interfaces. In case ②, the initial (*t* = 0) stress distribution shows a tensile stress at *z* < *z*_N_ and a compressive stress at *z* > *z*_N_ (Figure 4e). However, as it progresses, the greater removal of the monomer compared to ingression of the solvent within the boundary skin layers results in a transformation from tensile stress to compressive stress at (*z* < *z*_skin_), resulting in a rapid decrease of curvature. When the mass exchange continues, the transient “skin” effect subsides as the η profile becomes progressively uniform throughout the polymer cross-section, leading to the gradual decrease of stress magnitude throughout the polymer, resulting in a continuous increase of κ until it reaches κ_asymptote_, as shown in Figure 4e. This κ reversal is observed at *t* ≈ 10 s, which corresponds to the solvent first reaching the midpoint of the polymer network. While both skins experience a compression and thus shrink, this effect is greater at the leading edge of the front (*z*_f_ = 0.2 mm), yielding an initial decrease in κ followed by an asymptotic increase, as the bulk also experiences a compression, and thus yielding an overall “single fluctuation”. This behavior is observed for solvents such as acetone, ethanol, and toluene, as well as in isopropanol (albeit with a considerably higher asymptotic κ).

In case ③, the initial (*t* = 0) stress distribution shows a tensile stress at *z* < *z*_N_ and compressive stress at *z* > *z*_N_, as shown in Figure 4f. Both “skins” thus experience a net uptake and swell, which is however greater for the leading edge of the front. The first κ reversal corresponds to the solvent ingression reaching the midpoint, around *t* ≈ 5 s. The asymmetry in the kinetics of solvent uptake and monomer mass loss results in a transient overshoot of total mass, as shown in the inset of Figure 4c (and observed experimentally). At earlier times (*t* < 5 s), the pronounced and rapid ingress of the solvent compared to monomer removal at both interfacial skins results in a higher tensile stress at *z* < *z*_skin_ < *z*_N_ and lower compressive stress at *z*_N_ < *z*_skin_ < *z*, given the network asymmetry. The increase in the bending moment leads to a rapid increase in curvature. The transient effect is reversed by further solvent ingression, which leads to the homogenization of the swelling within the network, thus resulting in a rapid decrease of curvature. The second κ reversal occurs at *t* ≈ 100 s and is driven by network excess swelling (i.e., beyond its asymptotic value), which starts at *t* ≈ 50 s. Upon further extraction of the monomer, at a slower rate, the system tends to equilibrium; the overall network swelling is reduced, decreasing stress asymmetry throughout the polymer, which gradually increases κ until it reaches κ_asymptote_ (Figure 4f), governed by the intrinsic network asymmetry.

Employing our minimal model, we examine the coupling between solvent mass uptake and curvature fluctuation (detailed in SI Figure S7). As expected, increasing the mass diffusion rate of the monomer and solvent results in correspondingly faster curvature fluctuations. By contrast, varying the equilibrium solvent uptake *M*_solvent_(*t* → ∞) can qualitatively change the nature of the curvature fluctuation, from double to single to no fluctuation, and its value decreases.

### Underwater Propulsion by Asymmetric FPP Networks

We next consider the possibility of actuation or propulsion driven by the curvature fluctuations described above. We first select water as the solvent medium, supporting double curvature fluctuations of the PEDGA beams described above. Using 3D printing, we fabricate a “submersible” cylindrical structure, with appropriate buoyancy, and orthogonal “stabilizers” that rest at the water–air interface, preventing rotation along the long axis (shown in Figure 5a). A photopolymerized PEGDA slender beam (24 mm × 3 mm × 0.2 mm) is mounted transversally on the device, inserted onto a slit below the stabilizers. The asymmetric network beam is oriented such that the less cross-linked facet is oriented toward the “front” of the submersible, and thus, the conversion gradient opposes the intended direction of motion.

**Figure 5:**
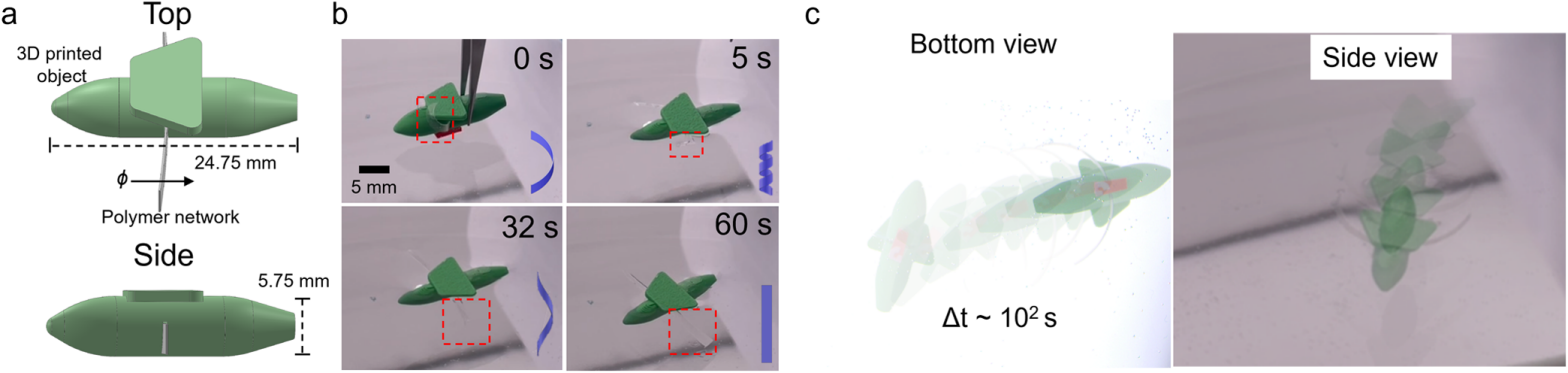
Demonstration of propulsion of a submerged object via curvature fluctuation. (a) Schematic of the submerged device fabricated by 3D printing, comprising a rectangular slit to accommodate an FPP network beam. The trapezoidal upper stabilizer rests at the surface of water and prevents rotation along the long axis of the device. (b) Initial response of an illustrative long FPP beam (40 mm × 3 mm × *z*_f_ = 0.2 mm) inserted into the submerged structure, from bending, coiling, and straightening the shape inserted into the floating object. (c) Device propulsion via curvature fluctuations, demonstrated in water (exhibiting double fluctuation). Supporting Video VO1 provided.

Figure 5b shows the submersible structure before (*t* = 0 s) and after water immersion (*t* > 0 s). At *t* = 0 s, we observe an initially bent beam, followed by rapid twisting of the coil on both sides, similar to other hydrophilic beams (i.e., liquid crystal elastomer) immersed in water.^39,40^ Subsequently, the twisted coil is slowly uncoiled and eventually flattened. Figure 5c shows the time-lapse propulsion from bottom and side views. In this illustration, propulsion does not occur in a straight direction, which is attributed to a slight length imbalance of the polymer beam across the centerline of the device. In this exploratory realization, the beam curvature fluctuation is able to propel the object in water, with a ∼5 cm net displacement. For completion, we also consider propulsion in ethanol (which exhibits only a single fluctuation) and redesign the submersible device to be compatible with a lower density medium. Compared to water, propulsion in ethanol results in shorter displacement distance, of approximately 2 cm, but with greater velocity (as detailed in SI Section 5). While this difference can partly be attributed to variations in beam bending amplitude between ethanol and water, additional hydrodynamic effects (due to viscosity or buoyancy differences) cannot be ruled out. Finally, the present demonstration of submersible propulsion relies on a single or double flap of an FPP beam, in contrast to the multiple autonomous snapping polymer gels reported by Kim et al.^7^ Our experimental configuration employs an unconstrained beam, yielding continuous, gradual motion rather than exploiting mechanical instabilities to achieve multiple snapping transitions.

## Conclusions

We demonstrate the role of the solvent medium in the spatiotemporal response of asymmetric network beams fabricated by FPP. For PEGDA, a hydrophilic and biocompatible prepolymer (extensively employed to fabricate hydrogels), polar solvents yield the most pronounced curvature fluctuation response. We have examined the role of asymmetric conversion of the cross-linked polymer network, solvent interaction, and interfacial mass transfer and connected these to asymmetric swelling–shrinkage and resulting curvature fluctuations. While the spontaneous emergence of curvature can be reasonably expected, and indeed observed, in gradient networks formed by directional solidification processes such as FPP, associated with stress asymmetries at opposed interfaces, fluctuation of curvature seems less obvious to us. We find that such fluctuations occur due to transient, imbalanced mass exchanges of the monomer and solvent across the opposed interfaces, causing changes in swelling/shrinkage and the resulting stress fluctuations. We emphasize that selective solvent immersion is generally intrinsic to the process of “development” of photopolymerised materials with prescribed patterns (to remove the uncross-linked monomer and excess surface monomer), including photoresists or 3D printing by UV curing. It is thus ubiquitous and practically important for a range of light-cured polymeric materials and composites.

To describe the emergence of curvature and this fluctuating behavior, we employ a minimal FPP model, which is coupled to a Fickian model of interfacial mass diffusion and stress profile variation across the gradient network during solvent immersion (or development). Based on our previous experimental and modeling work,^12,26^ we judiciously select a conversion profile ϕ­(*z*) and network thickness *z*_f_ that spontaneously exhibit curvature and systematically examine the role of the solvent “developer”. The model supports both swelling and shrinkage of the network depending on solvent affinity (in this PEGDA case, solvent polarity). This coarse-grained, descriptive framework captures the main features of the observed curvature fluctuation behavior upon solvent immersion. The model provides mechanistic insights and qualitatively captures the material response.

While our model can adequately describe correlations between solvent–monomer exchange and macroscopic κ trends, some behaviors cannot be rationalized (e.g., the high κ_∞_ observed for isopropanol). Indeed, our continuum framework lacks molecular-level information, such as specific solvent–polymer–monomer interactions, conformational changes of polymer chains within the network, or complex co-nonsolvency or cosolvency in mixed solvents, which can nontrivially impact outcomes. Furthermore, we have not explicitly mapped experimentally the spatiotemporal evolution of composition profiles of the ternary system or that of mechanical properties of the network. Our framework is thus primarily interpretative, rather than predictive and based on first principles. Furthermore, it can guide the design and engineering of environmentally responsive FPP actuators. To illustrate a potential practical application of these curvature fluctuations, we demonstrate the autonomous propulsion of a cm-sized device employing an FPP asymmetric network beam as an actuator. We believe that our experimental and theoretical framework can guide the design of a range of active materials based on asymmetric polymeric networks fabricated by directional solidification processes, with encoded actuation and locomotion.

## Supplementary Material







## Notes

Mass uptake, PEGDA monomer solubility experiment, Hansen solubility parameter estimations, curvature fluctuation amplitude analysis, and dynamic curvature modeling and parametrization (PDF)
Autonomous propulsion of a device equipped with an asymmetric FPP beam in water (MP4)
Autonomous propulsion of a device equipped with an asymmetric FPP beam in ethanol (MP4)
