# Supplementary material for: Curvature Fluctuations of Asymmetric Photopolymerized Networks: Impact of Solvent-Mediated Interfacial Exchanges
Source: ACS Appl Polym Mater. 2026 Jul 10;8(14):11048–57. doi: 10.1021/acsapm.6c00944 (PMC13410296; doi:10.1021/acsapm.6c00944)
Supplement: Supplementary file 1 [file ap6c00944_si_001.pdf]

Supplementary Information:

Curvature fluctuations of asymmetric photopolymerised networks: impact of solvent-mediated interfacial exchanges

Muhammad Ghifari Ridwan[a], Ghassan Sadaka[a], Mihai-Andru Angheliu[a], and João T. Cabral[a,*]

[a]*Department of Chemical Engineering, Imperial College London, London SW7 2AZ, United Kingdom*

***Email:** j.cabral@imperial.ac.uk

1 Assumption and calculation of mass uptake

The PEGDA films were fabricated with a thickness of ∼0.2 mm. To ensure consistent results across samples, all polymers were pad-dried before weighing to remove any solvent or unreacted monomer on the surface. The initial mass of each film was approximately 2.7 ± 0.2 mg, which corresponds to the dry polymer network and the trapped monomer present after curing. Each polymer was then immersed in a solvent for a defined period. For each solvent, measurements were performed at 10 s, 20 s, 30 s, 50 s, 1 min, 2 min, 3 min, 4 min, 5 min, and 2 h. After the specified time elapsed, the sample was removed, pad-dried again to eliminate excess surface liquid, and immediately weighed. This intermediate mass therefore, reflects the polymer network plus any solvent that diffused into it, in addition to the residual unreacted monomer. To obtain the mass of crosslinked polymer, all samples were subsequently immersed in its specific solvent and left to dry in air for two hours and then weighed again. Here, we assume that only the solvent will evaporate while PEGDA monomer remains. We verify this assumption by placing ethanol and PEGDA monomer in a cylindrical tube in ambient air. By measuring the mass changes over time, we confirm that ethanol evaporates, while PEGDA monomer does not (Figure S1, at comparable timescales. Across experiments and depending on the solvent, this baseline was consistently measured as 1.4 ± 0.2 mg, and this value was therefore taken as the mass of the fully cured PEGDA network with no monomer/solvent within the network.

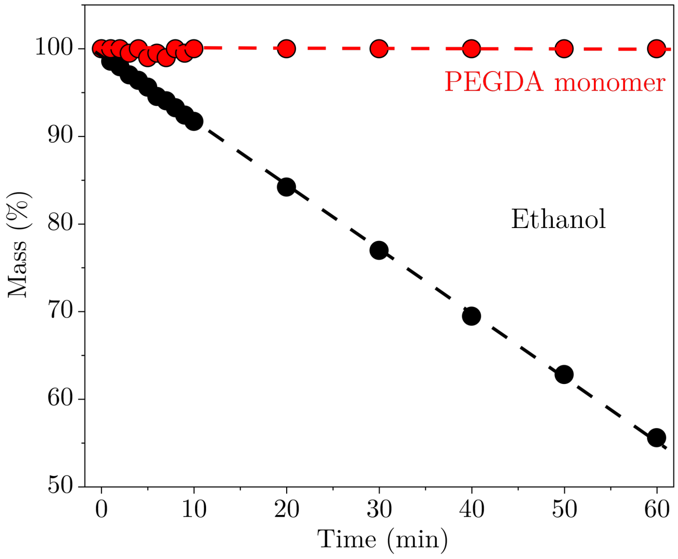

Figure S1 Mass measurement during evaporation of ethanol and PEGDA monomer (approximately 0.2 g) in air, at ambient conditions. The liquids are placed in a round cylinder container of 5 mm radius (and thus opening area of 78 mm^2) and allowed to evaporate atop a scale.

Here, we discuss the possible source of error in our measurements. First, upon the fabrication of the sample via UV irradiation, we remove the sample from printing substrate and pad-dried to remove excess unreacted monomer. Second, upon the solvent development stage, we pad-dried the sample to remove excess solvent. The two possible sources of error arise due to under-removal or excess removal of monomer or solvent. We anticipate this by presenting multiple measurements at each timestep, with different samples for each single data point. In addition, we found that there is a broad range of pad drying ('typical') conditions for which the mass measurement remains unchanged (pad is in contact with the outer parts of the polymer, hence mostly absorbing the excess on the surface), and the findings are thus repeatable and reproducible.

2 PEGDA monomer solubility experiments

To establish a baseline of PEGDA-solvent compatibility prior to photopolymerization, a simple miscibility test was performed with a range of solvents in addition to the solvent list in the main text. The list of solvents used can be found in Table 1. A $\sim$5 mL of each solvent were collected and transferred into clean glass vials. A small quantity of PEGDA monomer ($\sim$ 200 μL) was added to each solvent vial and mixed in a vortex mixer. The mixtures were then inspected visually before and after mixing to determine whether a homogeneous, single-phase solution had formed or whether phase separation was present. Solvents were therefore classified as "✓" if they completely dissolved the PEGDA monomer and resulted in a clear, single-phase mixture, or "$\times$" if phase separation was observed and the monomer remained insoluble.

Table S1 Miscibility of PEGDA monomer in different solvents, and Hansen solubility parameters for each solvent.

No.	Solvent	Dissolved Status	δD	δP	δH	δT
1	Heptane	×	15.3	0	0	15.3
2	n-Octane	×	15.5	0	0	15.5
3	Cyclohexane	×	16.8	0	0.2	16.8
4	Benzene	✓	18.4	0	2.0	18.5
5	o-Xylene	✓	17.6	1.0	3.1	17.9
6	Toluene	✓	18.0	1.4	2.0	18.2
7	Chloroform	✓	17.8	3.1	5.7	18.9
8	Chlorobenzene	✓	19.0	4.3	2.1	19.6
9	1-Pentanol	✓	16.0	4.5	13.9	21.7
10	THF	✓	16.8	5.7	8.0	19.5
11	Tetrachloroethylene	✓	18.3	5.7	0	19.2
12	1-Butanol	✓	16.0	5.7	15.8	23.2
13	Isopropanol (2-propanol)	✓	15.8	6.1	16.4	23.6
14	1,2-Dichlorobenzene	✓	19.2	6.3	3.3	20.5
15	1-Propanol	✓	16.0	6.8	17.4	24.6
16	DCM	✓	17.0	7.3	7.1	19.8
17	Ethanol	✓	15.8	8.8	19.4	26.5
18	2-Butanone (MEK)	✓	16.0	9.0	5.1	19.1
19	Acetone	✓	15.5	10.4	7.0	19.9
20	Ethylene glycol	✓	17.0	11.0	26.0	33.0
21	Glycerol	✓	17.4	12.1	29.3	36.2
22	Methanol	✓	14.7	12.3	22.3	29.4
23	N,N-Dimethylformamide	✓	17.4	13.7	11.3	24.9
24	Water	✓	15.6	16.0	42.3	47.8
25	Dimethyl sulfoxide (DMSO)	✓	18.4	16.4	10.2	26.7

A Hansen solubility analysis was conducted for PEGDA using solubility observations across 25 solvents, following the approach of Diaz et al.[1] To capture all good solvents within Hansen space, a two-sphere representation was required (Fig. S2a). For the first sphere, the fitted parameters were δD = 15.66 MPa$^{0.5}$, δP = 12.38 MPa$^{0.5}$, and δ_D = 30.72 MPa$^{0.5}$, corresponding to an overall solubility parameter δ_T = 36.63 MPa$^{0.5}$

and a Hansen radius R_o = 12.14. For the second sphere, the fitted parameters were δD = 17.85 MPa$^{0.5}$, δP = 6.88 MPa$^{0.5}$, and δH = 8.42 MPa$^{0.5}$, giving δT = 20.90 MPa$^{0.5}$ and R_o = 9.75. To enforce thermodynamic consistency, the fitting procedure additionally imposed that good solvents satisfy an estimated Flory–Huggins interaction parameter $\chi < 0.5$. The interaction parameter was linked to the Hansen distance via ($\chi = \alpha \frac{R_a^2}{4V_S RT}$),[2] using an empirically derived coefficient ($\alpha = 0.6$) reported by Lindvig et al. to reflect a broader range of polymer systems.[3] The solubility parameters obtained for the second sphere are in close agreement with previously reported values for Poly-ethylene glycol (PEG), both from empirical measurements and theoretical estimates, with only a minor deviation in the polar contribution.[4,5] In prior studies, a single Hansen sphere often fails to capture the solubility behaviour in highly polar and strongly hydrogen-bonding solvents, particularly water and lower alcohols; accordingly, a two-sphere representation is frequently adopted to account for these classes of solvents. For PEGDA, this dual behaviour can be rationalised by its molecular architecture, where the terminal diacrylate groups introduce interactions distinct from those of the PEG backbone. Similar multi-sphere descriptions have been reported for materials composed of moieties with contrasting solubility characteristics (e.g., block copolymers, ionic liquids, and surfactant-like systems), which are designed to bridge otherwise distinct solvent environments. In this interpretation, the more polar and hydrophilic sphere may be associated with acrylate-rich interactions, whereas the less polar, moderately hydrophilic sphere is more representative of the PEG chain segment. To this end, we select 8 solvents with different polar Hansen solubility parameters (S2b).

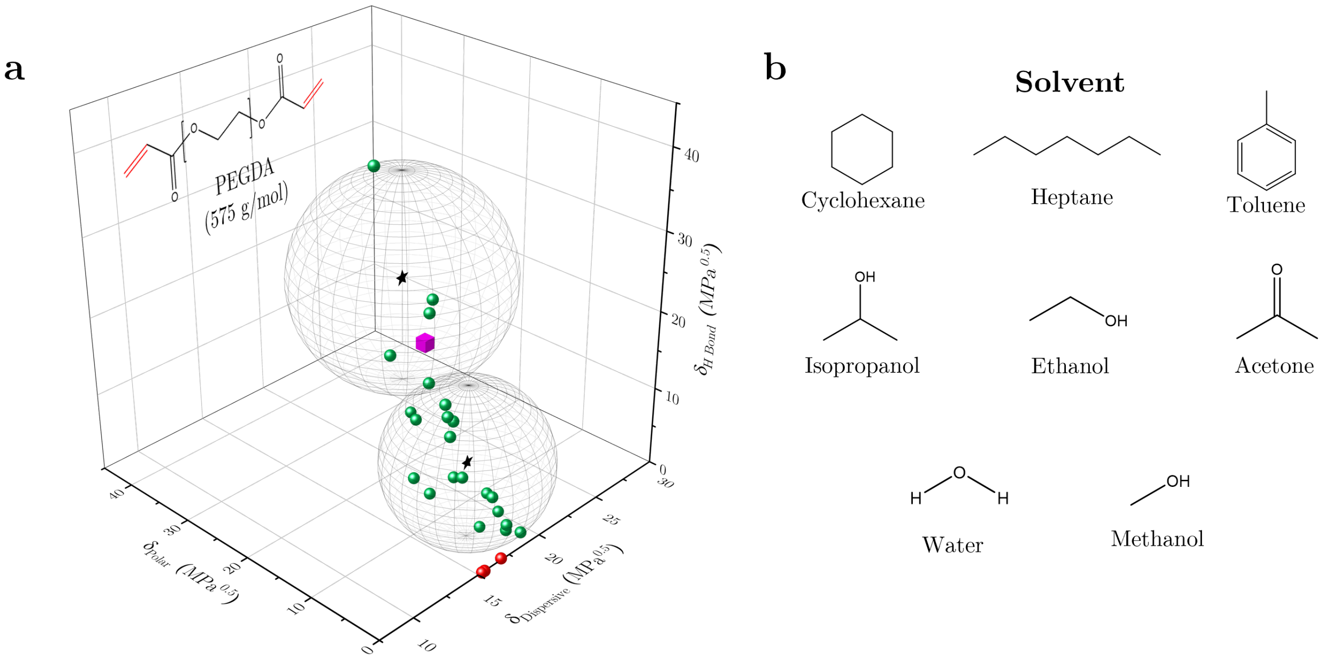

Figure S2 (a) Hansen spheres of PEGDA estimated for 25 representative solvents listed on table 1. (b) Molecular structure of solvents used for curvature measurement in the main text, representing a range from polar to non-polar solvents.

3 Amplitude analysis of fluctuating curvature

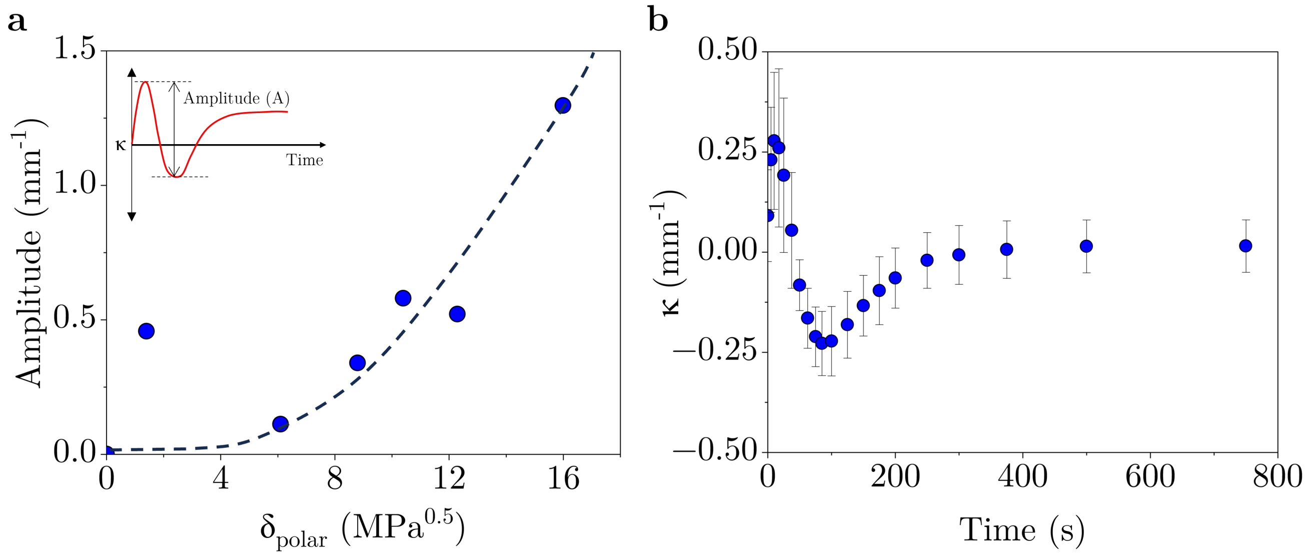

Figure S3 (a) Curvature amplitude versus Hansen polar solubility parameter. Inset: definition of the amplitude obtained from time-dependent curvature measurements. The dashed line is guide to the eye. (b) Demonstration of repeatability of curvature fluctuations of PEGDA asymmetric polymer network in ethanol, where the error bars are computed from 4 distinct experiments.

t

Here, we further analyse the time-dependent curvature data by quantifying the amplitude of the curvature fluctuations. The amplitude is extracted from the temporal curvature profile as defined in the inset of Fig. S3a. In addition, we also ensure the repeatability of the fluctuating behavior of κ in ethanol. We conduct 4 separate experiments and observed consistent trends across these. We then plot the resulting amplitude as a function of the Hansen polar solubility parameter (Fig. S3a) to examine the influence of solvent polarity on the mechanical response of the network. We observe a clear (exponential-like) correlation between the curvature amplitude and solvent polarity, indicating that the magnitude of the mechanical deformation increases systematically with increasing Hansen polar solubility parameter. Toluene appears as an outlier in this trend, which we interpret as arising from the challenges of the Hansen solubility framework to accurately describe systems governed by hydrogen-bonded interactions. Overall, this trend suggests that solvent–polymer interactions, governed by solvent polarity, play a dominant role in modulating the swelling-induced curvature response.

Our descriptive model presently lacks a molecular framework and thus a truly predictive nature. While solvent polarity serves as a useful indicator for curvature fluctuations, and our model can parametrise the behaviour in a self-consistent way, the observed curvature evolution undoubtedly arises from a combination of swelling thermodynamics, diffusion kinetics, and plasticisation effects. The well-known Flory-Rehner model provides a theoretical foundation to describe the swelling of polymer networks in solvent, as a competition of

two primary and competing effects: network mixing (generally described within a lattice, and a χ parameter) that favours mixing and solvent uptake, elasticity that resists swelling, driven by elastic retraction of the network. The FR model has a number of well-known limitations, including the assumption that networks are ideal and uniform. Due to the spatial variation in crosslinking density encoded during frontal photopolymerisation (FPP), the swelling ratio is inherently local, providing the structural basis for curvature generation. Our dynamic question involves a 3 component system (network, monomer and solvent) where the monomer is effectively a theta solvent and the solvent is generally 'good', but evidently involves several interaction parameters and effective diffusion coefficients. Diffusion also depends on molecular size, intermolecular interactions, and local network structure. A tighter mesh can restrict transport, which might be described by free-volume and transition-state descriptions of diffusion in polymers (e.g., Eyring-type models), where increased crosslinking reduces free volume and slows penetrant mobility. 'Monomer' length (effectively oligomer or pre-polymer) compared to the network mesh-size will also dictate which diffusion dynamics (e.g., Rouse or reptation) are relevant to describe transport. Evidently solvent uptake will enhance mobility in a complex spatiotemporal manner, as the diffusion coefficient can depend on the concentration field and network conversion. A molecular-level predictive theory must take into account the interplay of these various factors. Our present coarse-grained model provides a framework to interpret the experimental observations and guide the design of functional PEGDA networks.

4 Details of solvent-monomer exchange simulation on gradient polymer network

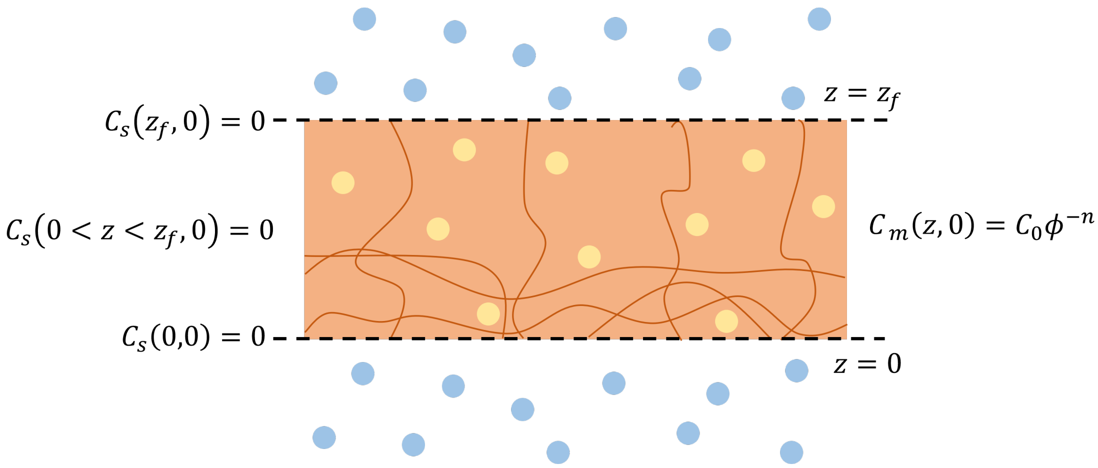

Figure S4 Schematic of simulation system with defined boundary and initial conditions, where the illuminated surface is defined at $z = 0$ and the beam thickness is at $z \equiv z_f$. Blue and yellow circles represent solvent and unreacted monomer.

To understand the dynamic behavior of asymmetric network during solvent immersion, we adapt our previous model[6,7] by incorporating diffusion exchange phenomena between solvent and monomer inside gradient network via Fick's 2nd law:

$$\frac{\partial C_m(z,t)}{\partial t} = \frac{\partial}{\partial z}\left(D_{e,m}\frac{\partial C_m(z,t)}{\partial z}\right) \tag{S1}$$

$$\frac{\partial C_s(z,t)}{\partial t} = \frac{\partial}{\partial z}\left(D_{e,s}\frac{\partial C_s(z,t)}{\partial z}\right) \tag{S2}$$

where $C(z,t)$ is the distribution of concentration in z-direction of the polymer network, D_e is an 'effective' diffusion constant, and $t_{immersion}$ is solvent immersion time. Subscripts m and s correspond to monomer and solvent parameters. We quantify D_e as a function of ϕ, $D_e = D_0\phi^{-1.5}$ (following[8]). To account the variability of solvent absorption capacity of asymmetric polymer network, we apply boundary conditions according to the mass uptake data:

$$\int_0^{z_f} C_m(z,t=\infty)\phi^{-1/3}dz = \frac{M_{total,m}(t=\infty)}{A} \tag{S3}$$

$$\int_0^{z_f} C_s(z,t=\infty)\phi^{-1/3}dz = \frac{M_{total,s}(t=\infty)}{A} \tag{S4}$$

where M_{total} and A is total mass and surface area exposed to solvent respectively. The surface area along z-

direction can be neglected when $z^2 << A$. The initial solvent and monomer concentration profile ($C_s(z,t=0)$ and $C_s(z,t=0)$) (Fig. S4) are build upon the results of solvent mass uptake ($M_{total,s}(t=0)$ and $M_{total,m}(t=0)$) experiments. It assumes a simple relation between conversion $\phi(z,t)$ and solvent/monomer uptake to solve this respective equation: $M(t=0) = \int_0^{z_f} C_0\phi^{-n}dz$, generating value of C_0. The initial distribution of monomer then is defined as $C(z,0) = C_0 \times \phi^{-n}$, where n is a parameter for polymer absorption capacity power law exponent.

To build relations between these physical phenomena with the mechanical response of the network, we introduced the reduced swelling/shrinking parameter termed *swell-shrink* ratio, $\eta_{swell-shrink} \equiv \frac{C_s(z,t)+C_m(z,t)}{C_m(z,0)}$, defined as the mass ratio of solvent and monomer to initial monomer concentration; the network has $\eta_{shrink-swell}$<1 in shrinkage state, and $\eta_{shrink-swell}$>1 when in swelling state.

To simulate the evolution of mechanical properties during mass exchange, we utilise the empirical relation between the Young's modulus (E) and strain (ε) against ϕ and $\eta_{shrink-swell}$. The strain to $\eta_{shrink-swell}$ relationship is as follow ,

$$\varepsilon(z,t) = [\varepsilon_{swell}(\phi) - \varepsilon_{dry}(\phi)]\eta_{shrink-swell}(z,t) + \varepsilon_{dry}(\phi) \tag{S5}$$

where ε_{swell} and ε_{dry} are defined based on Zhao et al.[9]

$$\varepsilon_{swell} = \left[\frac{g}{\phi^{0.6} - 1 + g}\right]^{1/3} - 1 \tag{S6}$$

$$\varepsilon_{dry} = [1 - h(1-\phi)]^{1/3} - 1 \tag{S7}$$

The minimum value of E is introduced to accommodate the sudden development of mechanical properties of solidified network at ϕ_c,[10] which we denote by E_0.[10] The evolution of the mechanical Young's modulus is empirically written as,

$$E(z,t) = E_0 + E_c(\eta_{shrink-swell})^{-n_1}(\phi - \phi_c)^{n_2} \tag{S8}$$

This empirical relation computationally allows the value of $\eta_{shrink-swell} > 1$ without returning error while maintaining the exponential trend in respect to η and ϕ.[7,9–11] To finally simulate the result, we compute numerically $C_m(z,t)$ and $C_s(z,t)$. We then compute $\eta_{shrink-swell}$, followed by $E(z,t)$ and $\varepsilon(z,t)$, and finally z_n and κ. Those parameters are calculated at pre-determined timestep and space ensuring numerical stability according to ϕ and η_{evap}. We then listed all data used in the simulation below:

Table S2 Parameters for dynamic curvature driven by solvent-diffusion simulation for $z_f \approx 0.2$ mm

No.	Parameter	Value	Unit
1	Irradiation dose, d	20.1	mJ/cm^2
2	Critical monomer-to-polymer conversion, ϕ_c	0.2	
3	Conversion rate constant, K	0.022	cm^2/mJ
4	Optical attenuation coeffcient, μ	3.4	mm^{-1}
5	Critical Young's modulus, E_C	100	MPa
6	Initial Young's modulus, E_0	0.05	MPa
8	Power law conversion, n_2	2.3	
9	Power law solvent, n_1	1.88	
10	Swelling constant, g	1.8	
11	Drying constant, h	0.6	
12	Solvent base diffusion constant, $D_{s,0}$	10×10^{-6}	mm^2/s
13	Monomer in water base diffusion constant, $D_{m,water,0}$	4×10^{-6}	mm^2/s
14	Monomer in ethanol base diffusion constant, $D_{m,ethanol,0}$	9×10^{-6}	mm^2/s
15	Monomer in heptane base diffusion constant, $D_{m,heptane,0}$	5×10^{-7}	mm^2/s

Figure S5 shows spatial distributions of solvent and monomer across the film thickness (Fig. S5). In case (i), the solvent concentration remains constant over time. In contrast, in cases (ii) and (iii), the solvent concentration increases progressively. On the unreacted monomer side, the concentration in case (i) likewise remains unchanged, whereas in cases (ii) and (iii) it increases with time.

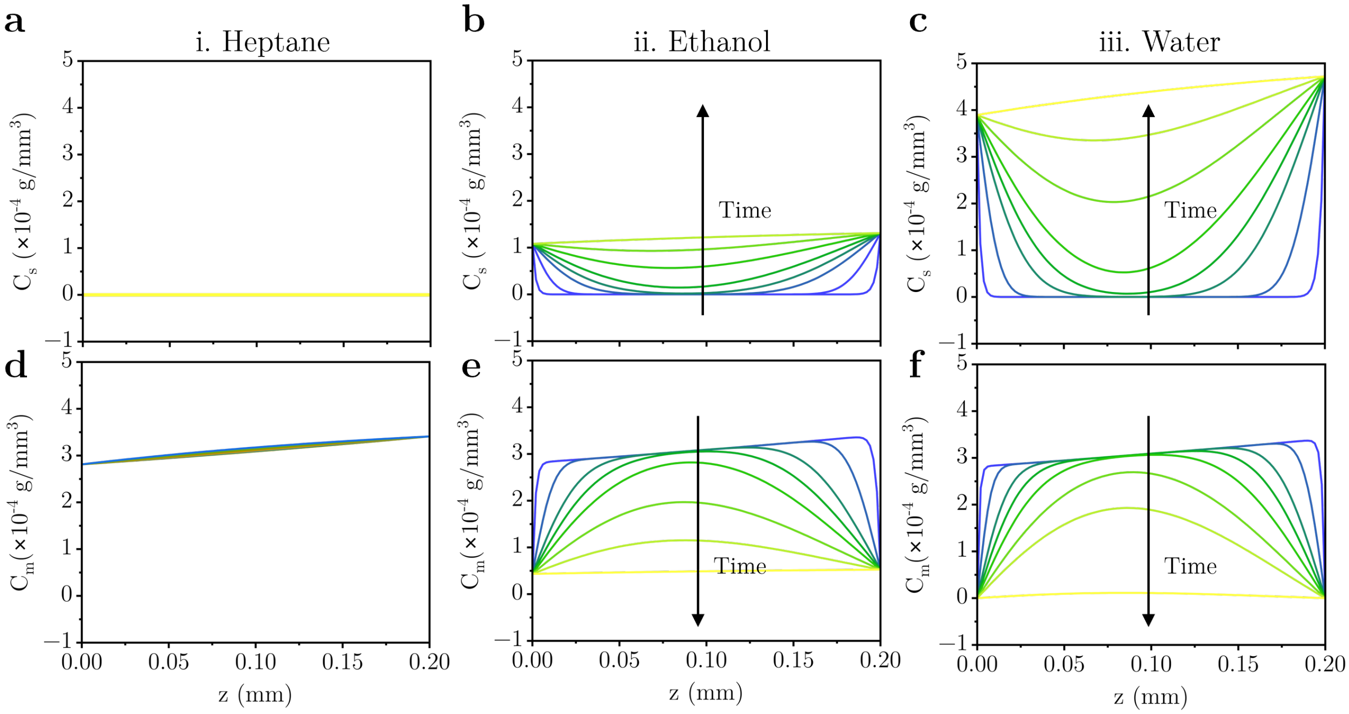

Figure S5 Simulation results of (a-c) solvent (C_s) and (d-f) monomer (C_m) concentration distribution for three different solvents: heptane, ethanol, and water.

Further, the stress (σ) is normalized with stress at neutral axis ($\sigma = E\varepsilon - E_{NA}\varepsilon_{NA}$). To this end, we provide the time evolution of mechanical properties based on the simulation below:

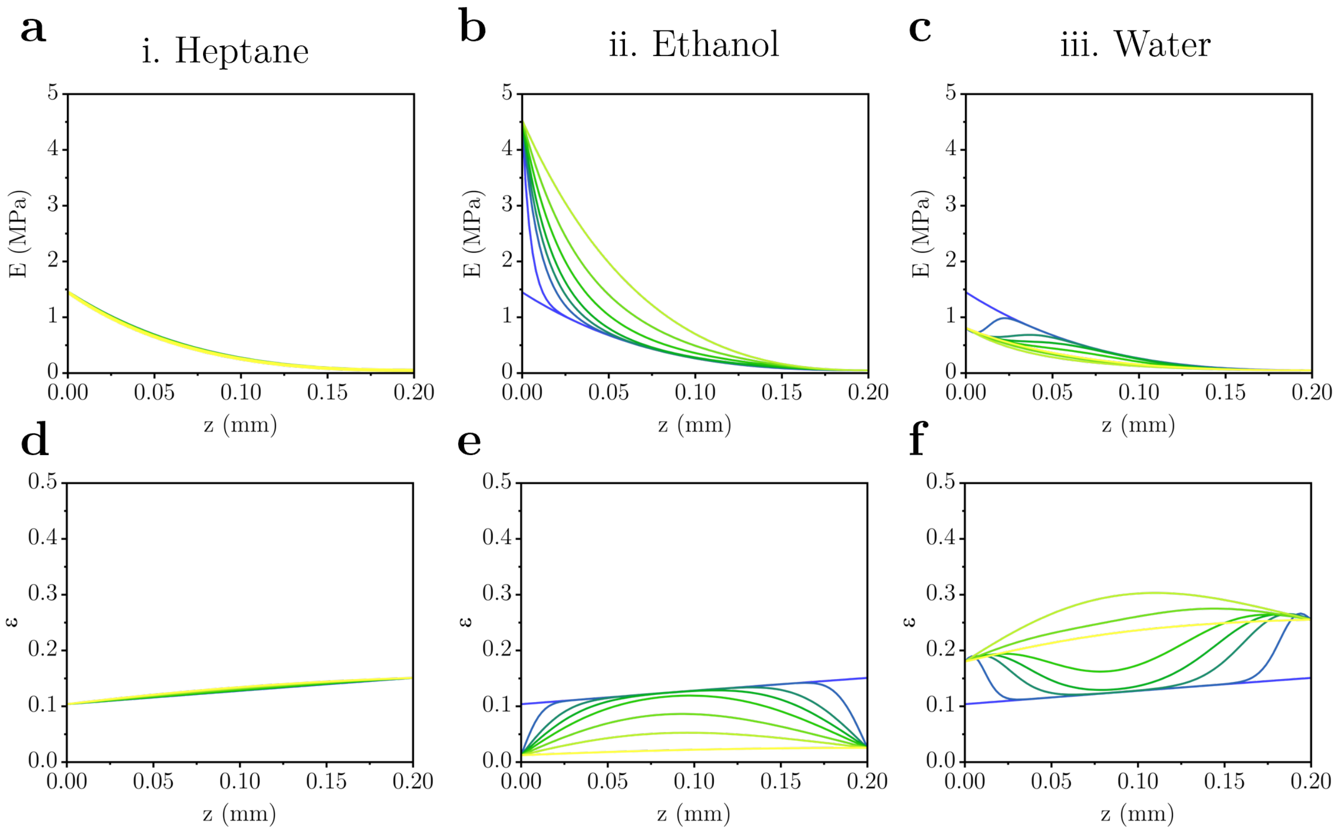

Figure S6 Time evolution of (a-c) Young's modulus and (d-f) strain for three different solvents: heptane, ethanol, water; across asymmetric network with z_f =0.2 mm^{-1}.

In case (i), there are minimal changes in the mechanical properties (E and ε) (Fig. S7a,d). In case (ii), the simulation results also exhibit a rapid increase of E near z=0 compared with $z = z_f$, indicating the exponential relationship between E and ϕ (Fig. S7b). In addition, the simulation results show that ε decreases significantly at the base ($z = 0$) and front ($z = z_f$) of the network (Fig. S7e). This strain distribution follows the $\eta_{shrink-swell}$ trend. Lastly, in case (iii), E is decreased over time due to the swelling process, and ε is increased as the volume increases (Fig. S7c,f).

To further investigate the influence of kinetic parameters on curvature evolution, we performed a series of parametric analyses focusing on the diffusion prefactor (D_0 for both solvent and monomer species) and the asymptotic mass, $M(t \to \infty)$. First, D_0 was varied by applying multiplicative factors of 0.5, 1, and 2. As expected, increasing D_0 accelerated the diffusion process, results in more rapid curvature fluctuations. However, these changes produced only minor variations in the relationship between the curvature, κ, and the total monomer fraction, $M_{\text{total,fraction}}(t)$. Next, $M(t \to \infty)$ was scaled by factors of 0.5, 1.3, and 2. For the cases of $1.3, M(t \to \infty)$ and $2, M(t \to \infty)$, curvature fluctuations remained evident throughout the evolution. In contrast, reducing the asymptotic mass to $0.5M(t \to \infty)$ substantially suppresses curvature fluctuations. Moreover, variations in $M(t \to \infty)$ produced pronounced changes in the relationship between κ and $M_{\text{total,fraction}}(t)$, indicating that the

asymptotic mass exerts a stronger influence on curvature behavior than the diffusion prefactor D_0.

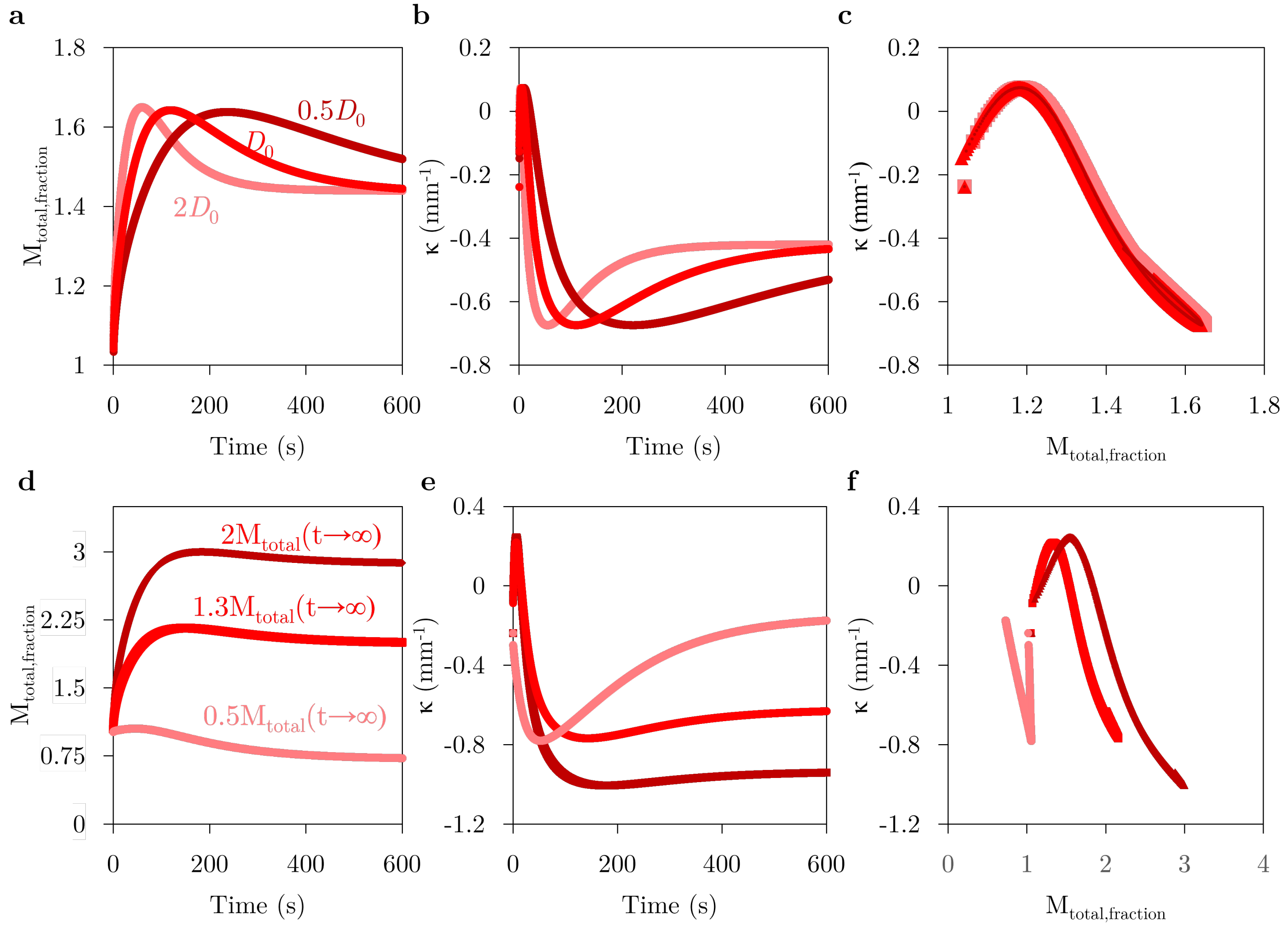

Figure S7 Impact of base diffusion constant, $D_{s,0} = 1 \times 10^{-5}$ and $D_{m,0} = 4 \times 10^{-6}$ mm^2/s, and equilibrium mass intake, m_∞ =0.0018 g on curvature fluctuations κ. (a) Evolution of $M_{total,fraction}$ and (b) resulting curvature (κ) over time; (c) relation between curvature (κ) and $M_{total,fraction}$ with variation of 0.5D_0, D_0, and 2D_0. (d) Evolution of $M_{total,fraction}$ and (e) resulting curvature κ as a function of time; (f) relation between curvature (κ) and $M_{total,fraction}$ with variation of M_∞, $1.3 \times M_\infty$, and $2 \times M_\infty$.

5 Propulsion in ethanol

Here, we provide experimental part of the floating device that propels forward due to the curvature changes of asymmetric FPP network. To enable the floating device in a lower-density medium than water (i.e., ethanol), we change the material of floating device from poly-lactic acid (density of ~ 1.24 g/cm^3) to acrylonitrile butadiene styrene (density of ~ 1.05 g/cm^3) and change the design to ensure appropriate floatation. In comparison with water, the propulsion in ethanol yielding lower total displacement (Fig. S8a). In contrast, the overall displacement rate, v, in ethanol is higher than in water (Fig. S8b,c). While these contrasting phenomena can be correlated with the amplitude of fluctuation, we acknowledge that this also could be affected by the hydrodynamics and buoyancy effects of the system, which will be separately investigated.

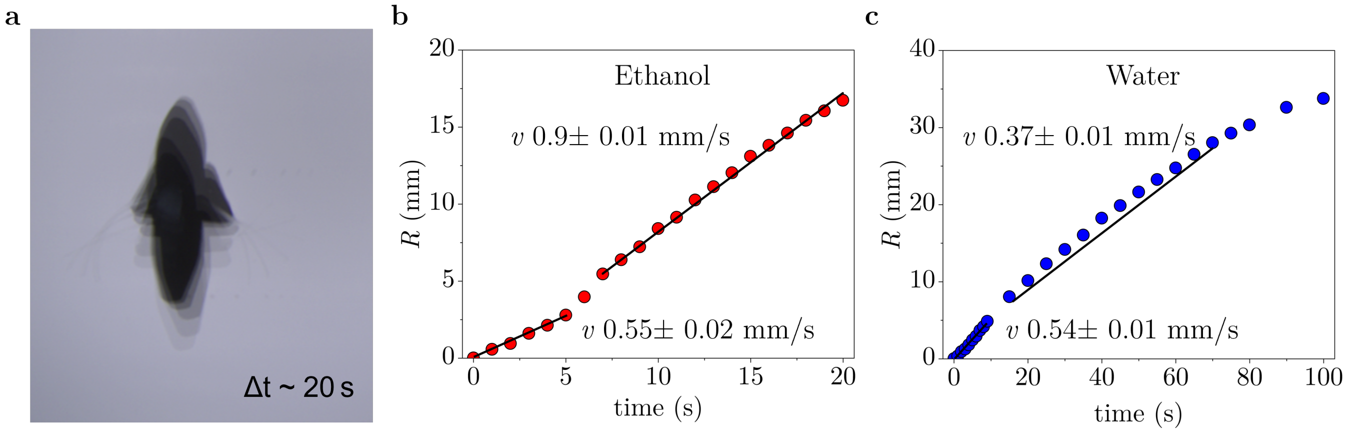

Figure S8 Illustrative displacement a floating device immersed in ethanol medium, showing slower propulsion within a 20 s time interval (compared to that in water, shown in the main paper). Supplementary Video VO2 provided.

References

[1] M. Díaz De Los Ríos and E. Hernández Ramos, *SN Applied Sciences*, 2020, **2**, 676.

[2] *Hansen Solubility Parameters* , `https://hansen-solubility.com/`, Accessed: 2026-02-26.

[3] T. Lindvig, M. L. Michelsen and G. M. Kontogeorgis, *Fluid Phase Equilibria*, 2002, **203**, 247–260.

[4] S. Thakral and N. K. Thakral, *Journal of Pharmaceutical Sciences*, 2013, **102**, 2254–2263.

[5] C. Özdemir and A. Güner, *European Polymer Journal*, 2007, **43**, 3068–3093.

[6] M. G. Ridwan, Z. Ahmad, A. Vitale and J. T. Cabral, *Advanced Materials Interfaces*, 2026, e00879.

[7] M. G. Ridwan, H. M. Dizman, I. Bentley, A. Vitale and J. T. Cabral, *Macromolecules*, 2026, acs.macromol.5c02783.

[8] H. Tokuyama, Y. Nakahata and T. Ban, *Journal of Membrane Science*, 2020, **595**, 117533.

[9] Z. Zhao, J. Wu, X. Mu, H. Chen, H. J. Qi and D. Fang, *Macromolecular Rapid Communications*, 2017, **38**, 1600625.

[10] J. Wang, X. Mu, D. Li, C. Yu, X. Cheng and N. Dai, *Advanced Engineering Materials*, 2019, **21**, 1801279.

[11] Z. Zhao, J. Wu, X. Mu, H. Chen, H. J. Qi and D. Fang, *Science Advances*, 2017, **3**, e1602326.
